# Neural crest cell genes and the domestication syndrome: A comparative analysis of selection

**DOI:** 10.1371/journal.pone.0263830

**Published:** 2022-02-11

**Authors:** Andrew O. Rubio, Kyle Summers

**Affiliations:** Department of Biology, East Carolina University, Greenville, NC, United States of America; Tokai University School of Medicine, JAPAN

## Abstract

Neural crest cell genes control the migration of neural crest cells to multiple parts of developing vertebrate embryos. A recent hypothesis posits that the “domestication syndrome” characteristic of domesticated animals is driven by selection for tameness acting on neural crest cell genes, particularly those affecting cell migration. This is posited to explain why this syndrome involves many disparate phenotypic effects. These effects can be connected to deficits in neural crest cell migration. This hypothesis predicts that patterns of selection on these neural crest cell genes will differ between domesticated species and related wild species. Specifically, it predicts higher levels of positive selection on these genes in domesticated species, relative to closely related wild species. Here we test this prediction in a comparative framework. We obtained DNA sequences from a public database (NCBI) for eleven key neural crest cell genes from a set of thirty domesticated vertebrates and matched close relatives that remain wild. We used the program Contrast-FEL in the software suite HyPhy to compare the number of sites under positive selection (as measured by non-synonymous to synonymous nucleotide substitution rates across codons) between these two types of taxa in a phylogenetic framework. We found that domesticated lineages showed a consistently higher level of positive selection on these key genes, relative to their closely related wild counterparts. In addition, we found support for relaxation of selection and purifying selection. We argue that this result is consistent with an important role for these genes in the domestication syndrome.

## Introduction

The “domestication syndrome” refers to an apparently disconnected set of phenotypic traits that appear to characterize domesticated species, in comparison with their wild relatives. In addition to tameness (reduced reactive aggression), these traits include other elements of behavior (such as prolongation of juvenile behavior), and aspects of morphology (brain and tooth size, ear and tail size and shape, craniofacial structure), and physiology (levels of andrenocorticotropic hormones and neurotransmitters, frequency of estrus cycles). The association between domestication and a seemingly unconnected suite of characters was noted and investigated by [1], but continues to be a topic of scientific debate to the present [2–4].

Wilkins et al. [2] have proposed a novel theory that purports to explain why all of these seemingly disparate features are connected. They take as their starting point the assumption that selection for tameness is the *sine qua non* of domestication: the idea that all successful instances of domestication involve the cumulative effects of selection for reduced aggression, generation after generation for a prolonged period. Given this assumption, the key question becomes: why does selection on this aspect of behavior affect a broad yet specific set of traits, including behavior, morphology, and physiology?

The answer, according to Wilkins et al. [2], is that selection for tameness acts specifically on genes that affect the formation, differentiation and migration patterns of neural crest cells (hereafter referred to as “neural crest cell genes”), especially genes influencing the migration of neural crest cells. Neural crest cells affect a wide variety of phenotypic traits, and the set of traits that they affect bears a remarkable resemblance to the set that is characteristically affected by the domestication syndrome. This includes well-known characteristics such as floppy ears and blotchy coloration, in addition to hormonal changes and reduced brain size. The suite of changes associated with the domestication syndrome appear to be consistent with a process of neoteny, and this may be associated with reduced quantity, impaired migration, or slower proliferation of neural crest cells [2, 5, 6].

Wilkins et al. [2] note that few of the traits characteristic of the domestication syndrome are found in all domesticates. It is likely that characteristics specific to each lineage affect whether a particular trait will be modified in domesticates relative to their wild counterparts. For example, lineages that already have short faces (e.g. felines) may show less reduction in facial projection (muzzle length) relative to lineages originally characterized by long muzzles (e.g. wolves compared to dogs). Nevertheless, Wilkins et al. [2] argue that changes in neural crest cell migration are likely to underlie differences in the morphology, physiology, and behavior across the wide variety of domesticated vertebrates [6].

The effects of these genes are very important from a medical perspective, as knockouts and mutant versions of these genes produce a plethora of serious medical conditions. These conditions have long been known to the medical community, and have been designated as “neurocristopathies” [7]. A wide variety of diseases fall into this category, including Waardenburg Syndrome, Hirschsprung Disease, Ondine’s Curse, and multiple sclerosis [8, 9], among many others. Hence, a unified theory of the underlying selection pressures driving the domestication syndrome should be highly valuable in attempts to understand the underlying basis for our susceptibility to a wide variety of neural crest cell gene-related diseases.

Recently, it has been proposed that the domestication syndrome applies in important ways to *Homo sapiens*, in that humans are a “self-domesticated” species. Self-domestication has been posited to affect a wide variety of emotional and behavioral traits in the human lineage, including the evolution of cooperation and the evolution of language [6, 10–12].

Wilkins et al. [2] review a compelling array of evidence that mutations in these genes are associated with an array of phenotypic effects that span the diversity of traits known to be affected by the domestication syndrome (they note that such “mutations” could involve copy number changes or even epigenetic changes, in addition to standard point mutations). They note that the number of neural crest cell genes underlying the domestication syndrome is likely to be large (i.e. the causes are likely to be polygenic), but do provide a list of candidate genes highly likely to be involved in the syndrome (based on genetic studies of haploinsufficiency effects and epistatic properties).

In this study, we attempted to address a major prediction of the neural crest cell gene hypothesis proposed by Wilkins et al. [2]: that the key neural crest genes should be under positive selection in domesticated species, relative to closely related wild species. As noted, Wilkins et al. [2] provide a list of neural crest genes that are likely to play crucial roles in the evolution of the domestication syndrome. As the authors note, this list is not comprehensive, but it does provide a set of genes that can be used to test the general prediction.

## Methods

### Choice of genes and taxa

**Fig 1:**
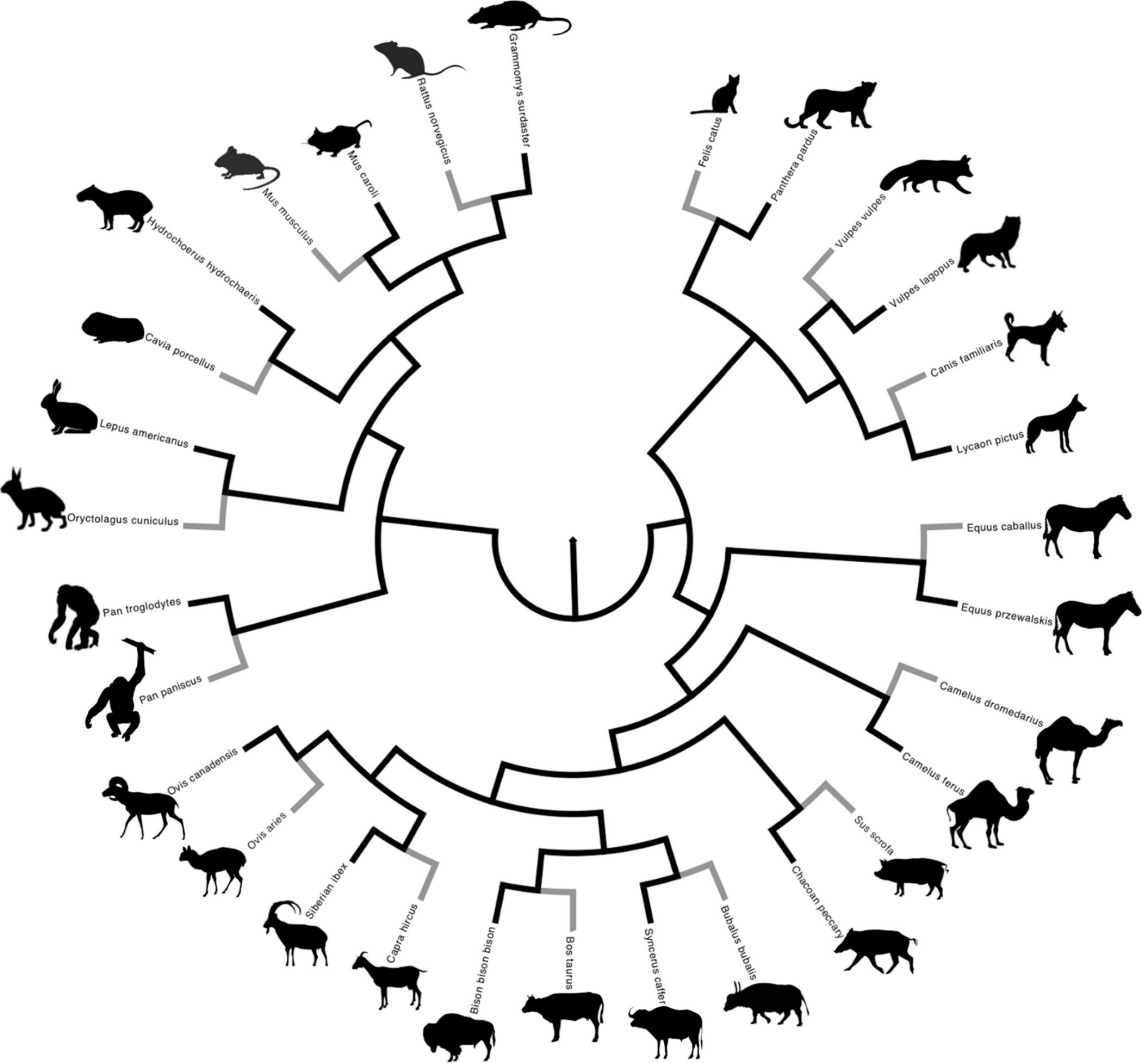
An evolutionary tree representing the 15 domesticated mammals (indicated with gray terminal branches) and their closely related wild relatives (indicated with black terminal branches) that were studied. The tree is based on multiple genes, as described in Upham et al. 2019. The tree was obtained as a “phylogeny subset” from the VertLife website (https://vertlife.org/data/). See text and S2 Table for species names. All mammal silhouettes were taken from http://phylopic.org and are under a public domain license. The pertinent information concerning each silhouette is available as a S1 File).

We chose to analyze most of the candidate genes listed in Table 2 of Wilkins et al. [2] with the exception of four color-pattern related genes which we suspected might be under positive selection for reasons unrelated to domestication (because similar genes have been found to be rapidly evolving in other systems). This left us with eleven candidate genes to analyze (S1 Table). We chose to restrict our analyses to mammalian taxa, as this group contains the vast majority of domesticated species, and taxa in this clade share basic physiological and morphological systems. This made it less likely that large differences in physiology or morphology (unrelated to domestication) would confound our ability to carry out meaningful comparisons. We selected common, well-known domesticated species of mammals (house mouse, Norway rat, guinea pig, rabbit, sheep, goat, pig, cattle, water buffalo, horse, dog, cat, (domesticated) red fox, and Arabian camel). We also included the bonobo, the sister species of the chimpanzee, which is thought to be self-domesticated [10]. For each of these taxa, we chose a closely related wild species for which we could obtain gene sequence data for each of the neural crest cell genes analyzed (Ryukyu mouse, African woodland thicket rat, capybara, snowshoe hare, bighorn sheep, Siberian ibex, chacoan peccary, plains bison, African buffalo, Przewalski’s horse, African wild dog, leopard, arctic fox, wild Bactrian camel, chimpanzee). These pairs of taxa (each domesticate and a closely related wild species) formed the basis for our comparative analysis, in a phylogenetic context. Fig 1 shows the phylogenetic tree of relationships of the taxa included in this study, which is based on Upham et al. [13].

### Collecting and aligning sequences

We obtained protein and nucleotide sequences from eleven neural crest cell genes and eleven control genes (S1 Table) from each of 15 domesticated mammal species and their closely related wild relatives (S2 Table) from GenBank (https://www.ncbi.nlm.nih.gov/genbank/). We were unable to find sequence references for a couple of non-domesticated mammals in GenBank. In order to retrieve these sequences, we used BLAST (Basic Local Alignment search) [14] to perform tblastn searches using protein sequences from their closely related domesticated relatives as a query against the entire genome of the non-domesticated animal in question for each gene. We compiled all nucleotide regions in numeric order from a single tblastn result output. We used ExPASy Translate [15] to translate nucleotide sequences into a protein sequence.

We used MAFFT [16] to align proteins from domesticated and non-domesticated mammals for each gene. Once protein sequences were aligned, we used PAL2NAL [17] for each gene to construct a multiple codon alignment from multiple sequence alignments of protein and nucleotides.

### Selection analyses

To analyze patterns of selection, we took a comparative approach. First, we obtained a phylogenetic tree (Fig 1) for the 30 species of mammals investigated with a topology based on up-to-date (maximum likelihood and Bayesian) methods of DNA sequence data analysis [13], using the interactive phylogeny subsets function for the mammalian phylogenetic tree available on the VertLife website (vertlife.org/data: [13]). This subset was downloaded on July 25^th^, 2020. We then used the Mesquite, version 3.70, suite of programs [18] to create a phylogenetic tree topology connected to the DNA sequence alignment specific to each gene. Note that in some cases we were not able to obtain reliable sequence data for all of the 30 species in the original set of mammals. In these cases we trimmed taxa from the tree from VertLife to match the reduced number of taxa in the alignment. Trimming was done in pairs, such that if one member of a pair was missing (either domesticated or wild species), the other member of that pair was also removed. The tree was saved in Newick format, following the sequence alignment (in fasta format), in a text file.

Analyses of selection were carried out using the HyPhy suite of programs [19]. Our main prediction concerned positive selection. Following the arguments presented in Wilkins et al. [2], we predicted that the candidate neural crest genes would experience positive selection more frequently or intensely in domesticates than the same gene in closely related wild species. In order to test this, we used the ContrastFEL program in the HyPhy software suite (www.hyphy.org; [19] via the Datamonkey2.0 server (http://www.datamonkey.org; [20]) and by using the program on a local computer (obtained via download)). The FEL (Fixed Effects Likelihood) program implements a maximum likelihood-based algorithm that detects the action of selection on individual codons, assuming that selection pressures are consistent throughout the phylogeny [19]. The algorithm optimizes branch lengths and nucleotide substitution parameters, then estimates nonsynonymous (dN) and synonymous (dS) substitution rates at each site under a complex model of substitution (MG94xREV) that specifies transition rates between each type of codon, and controls for transition/transversion rates, nucleotide mutation biases and equilibria, and codon equilibrium frequencies. A likelihood ratio test (LRT) is used to test for statistically significant evidence for selection on specific codons. The Contrast-FEL program [21] employs the FEL algorithm to compare sets of branches in a phylogeny, where different lineages have been consistently exposed to different environments. In our case, the domesticated lineages have clearly been under distinct environmental regimes compared to closely related wild lineages. The method tests whether selection has acted differently between the two different sets of lineages, in a codon-specific manner. In our study, we compared the number of sites (codons) where the dN/dS ratio was significantly higher (providing evidence for stronger positive selection) in the domesticated lineages compared to the wild lineages, and vice-versa, for each gene.

We also selected a set of “control” genes for the same (domesticate versus wild) comparisons, to determine whether differences between the domesticated and wild species were more pronounced for the neural crest genes relative to other genes. The control genes were selected in an *ad hoc* fashion by using the term “metabolism” in a search under the “Gene” category (restricted to mammals) on the NCBI website (https://www.ncbi.nlm.nih.gov/gene). We avoided genes known to be connected to traits affected by neural crest cells.

We also compared the evolution of the neural crest genes in the domesticated lineages to their wild counterparts using the RELAX program in HyPhy [22]. The algorithm in this program can identify the relaxation of selection in one designated set of branches (domesticates, in our case) compared to another (wild, in our case). It is also able to infer differences in the strength of purifying selection between these sets of branches. These comparisons were not directly linked to the predictions made by Wilkins et al. [2], but were of general interest, in our opinion.

## Results

Our results are consistent with the hypothesis that neural crest genes are under positive selection specifically in domesticates. The Contrast-FEL analyses revealed that, for 10 of the 11 neural crest genes, positive selection was significantly higher in domesticates than wild species for more codon sites at the p < 0.05 significance level (Table 1). In fact, we only identified one case where the dN/dS ratio was higher at a codon site in a gene in the wild species (*chd7*), and the vast majority (14) showed the opposite pattern for this gene. There was also one gene (*magoh*) where no significant differences in dN/dS were detected at any codon site in the neural crest cell genes.

**Table 1:** ContFEL: Number of codon sites in domesticates (D) compared to wild relatives (W) that had significantly higher dN/dS (p < 0.05).

	Gene	ContFEL	Relax	PurSel	PurSel2
**NEURAL CREST GENES**	***baz1b***	**D(5) > W(0)**	**NS**	**NS**	
	***sox10***	**D(5) > W(0)**	**Sig (p < 0.001)**	**NS**	**K = 0.10, LR = 22.35**
	***sox9***	**D(9) > W(0)**	**NS**	**NS**	
	***sox2***	**D(22) > W(2)**	**NS**	**NS**	
	***fgf8***	**D(1) > W(0)**	**NS**	**NS**	
	***Kit***	**D(14) > W(0)**	**NS**	**Sig (p = 0.018)**	**K = 8.95, LR = 5.58**
	***Gdnf***	**D(2) > W(0)**	**NS**	**NS (p = 0.061)**	**K = 50.00, LR = 4.15**
	***tcof1***	**D(75) > W(0) **	**NS**	**NS**	
	***chd7***	**D(27) > W(0)**	**NS**	**NS**	
	***foxd3***	**D(40) > W(0)**	**NS**	**NS**	
	***magoh***	**D(0) = W(0)**	**NS**	**NS**	
**CONTROL GENES**	***nme1***	**D(3) > W(0)**			
	***ube2d***	**D(0) = W(0)**			
	***ube2i***	**D(0) = W(0)**			
	***ern1***	**D(0) = W(0)**			
	***bace1***	**D(3) > W(0)**			
	***hmga2***	**D(0) = W(0)**			
	***commd1***	**D(4) > W(0)**			
	***opa2***	**D(0) = W(0)**			
	***fam3a***	**D(0) = W(0)**			
	***idh1***	**D(0) = W(0)**			
	***pon1***	**D(0) = W(0)**			

Relax = presence of relaxation of selection on domesticates relative to wild species. PurSel = presence of more intense purifying selection on domesticates relative to wild species. PurSel2: K statistics and likelihood ratio (LR) for tests of purifying selection/relaxation of selection. NS = Not Significant.

Our results also indicated that the signals of positive selection we found are specific to neural crest genes, and are not found in most of the control genes we used for comparison. A few of the control genes did show a pattern where positive selection was significantly higher at more codon sites at the p < 0.05 level in domesticates relative to wild species. However, a higher proportion of these genes (8/11 compared to 1/11) showed no significant difference between the domesticates and the wild species at any codon site. A pairwise nonparametric (Wilcoxon signed Ranks-Test) comparison between the number of sites showing significant evidence for positive selection in domesticates vs. wild species across the gene dataset showed a significant difference for the neural crest genes (Wilcoxon Signed-Rank Test for paired (domesticate vs. wild) samples, N_NCC_ = 11, Median N_NCC_ = -9, U = 16.50, Z = 2.61, p = 0.009). In contrast, the same test applied to the control genes showed no significant difference in positive selection between domesticates and wild controls (N_c_ = 11, Median N_c_ = 0, Z = 1.36, p = 0.174).

The test for relaxation of selection and purifying selection on the neural crest genes revealed one gene with significant relaxation of selection (*sox10*), one gene (*kit*) that showed significant intensification of purifying selection on the domesticates, and one gene (*gdnf*) that showed marginally nonsignificant (p = 0.061) evidence for purifying selection on domesticates.

## Discussion

Our analyses of positive selection using the Contrast-FEL program indicate that in almost all cases in our sample of eleven neural crest genes, there are typically multiple codon sites showing significantly higher dN/dS ratios in domesticates relative to wild species, but not the reverse. This evidence is consistent with the prediction that these genes have evolved under positive selection in domesticated lineages, as predicted by the hypothesis that these genes are under selection for tameness during the process of domestication [2].

Our analyses of the control genes showed that a small proportion have experienced positive selection at some sites in the domesticates, but not in the wild species. However, the comparison of the numbers of sites under selection between the neural crest cell genes and the control genes revealed that the neural crest cell genes showed higher numbers of sites under positive selection, relative to the control genes. It is perhaps not surprising that some genes show some evidence for positive selection in lineages that have been under intense selection for domestication for thousands of generations (in many cases). Yet our analysis supports the prediction that neural crest genes are especially likely to show signals of positive selection in domesticates, consistent with the hypothesis that strong selection for tameness impacted the neural crest cell genes in particular [2].

A number of other studies have compared domesticates with closely related wild species to search for signals of selection using genomic data (e.g. [23] (horses); [24] (cats); [25] (rabbits); [26] (dogs)). As reviewed in [27], these studies generally support the neural crest cell hypothesis, as they each discovered some neural crest cell genes that show signals of positive selection in the context of domestication. However, our study is (to our knowledge) the first to attempt a comparative analysis of selection on a sample of neural crest cell genes across a broad sample of domesticate-wild species pairs in a comparative context. Our results support the hypothesis that the evolution of domesticated lineages has involved convergent patterns of selection on a specific set of loci (the neural crest cell genes).

Our approach could be considered to provide a conservative test of the neural crest cell gene hypothesis. First, in the genomic analyses carried out on specific domesticates, most of the signals of selection are associated with regulatory regions, not coding sequences [27]. Hence, by focusing exclusively on selection on coding regions (as in our analyses), we excluded the most promising regions to find a signal. Second, the neural crest cell hypothesis does not require that the same set of neural crest cell genes will be affected during the process of domestication in all domesticated species [25]. The hypothesis proposes that a diverse set of neural crest cell gene mutations are likely affected during domestication (the hypothesis predicts a polygenic basis for the traits involved), and the key set of variant neural crest cell genes could well vary from domesticate to domesticate. Hence, our approach again provides a conservative test of the hypothesis.

As noted in the introduction, the neural crest cell genes analyzed here are known to be of medical relevance in humans. Recent evidence has revealed important connections between human health and the evolution of key human traits that are mediated by changes in neural crest cell genes. For example, the *baz1b* gene is strongly associated with Williams-Beuren syndrome. This gene has recently been shown to be a master regulator of the expression of multiple neural crest genes in humans, affecting craniofacial morphology and other features associated with the domestication syndrome [28]. This discovery, combined with close correspondence between the effects of the *baz1b* gene (and its downstream target genes), and key gene sets found to differ between modern and archaic humans in paleogenomic studies, provided strong support for the self-domestication hypothesis as applied to human evolution [28]. Variation in a number of other neural crest cell genes have also been found to underlie various human pathologies that may be connected to self-domestication (e.g. schizophrenia: [29]).

The fact that our analyses with the RELAX program showed several neural crest cell genes under purifying selection should perhaps not be surprising. In most cases, domestication is likely to involve the culling of individuals that do not show desirable traits from the stock population. This should impose strong purifying selection on the population(s) under domestication. The single gene that showed some evidence for relaxation of selection may be consistent with Darwin’s original argument [1] that domesticated animals experience less harsh conditions than their wild forebears. We note that because the models employed in these methods are codon-specific, there is no necessary contradiction in finding both positive and negative selection (or relaxation of selection) acting on the same gene.

The results of our HyPhy analyses, including the specific codon sites and substitutions under selection detected in our analyses are preserved in JSON files that are included in the supplementary materials. These may be useful to researchers interested in pursuing the molecular mechanisms whereby selection for tameness impacts specific aspects of neural crest cell gene function and interaction with other genes. Genetic manipulations (e.g. with CRISPR-Cas9) could be used to identify the specific effects of these nucleotide substitutions on the phenotype.

## Conclusions

In this study, we have tested a major prediction of the neural crest cell gene hypothesis for the evolution of the domestication syndrome, as proposed by Wilkins et al. [2]. This prediction was that neural crest cell genes would show strong signals of positive selection in domesticated lineages (relative to closely related wild lineages). Further, the hypothesis predicted that this pattern would be specific to neural crest cell genes, and would not be characteristic of other genes. Our results supported both of these predictions: 1) Significant evidence for positive selection was found at multiple codon sites in most domesticated lineages, but few or no sites in their wild counterparts. 2) The number of codons showing positive selection was significantly higher in domesticated lineages (relative to their wild counterparts) in the neural crest cell genes, but not in the control set of genes.

## Supporting information

S1 Table Neural crest cell and biochemical-molecular function genes. List of neural crest cell and control genes. First column indicates the names of neural crest genes investigated and the second column indicates the biochemical-molecular function of the gene.
(DOCX)

S2 Table Fifteen domestic species and their wild counterparts. First column indicates names of domesticated animals and the second column indicates their wild counterparts. NCBI RefSeq genome accession ID is listed for each species.
(DOCX)

S1 File Fig 1 mammal silhouettes. All mammal silhouettes used in Fig 1 were taken from Phylopic (http://phylopic.org) and are available for use under a Public Domain license. Work under this particular license is free to use without restrictions under copyright law (https://wiki.creativecommons.org/wiki/Public_domain)”. Below you will find the link to each mammal silhouette used, indicating their Public Domain license. Of all these silhouettes, one was further modified by an author of this manuscript, Andrew O. Rubio. Modification was done to silhouettes number 19 (http://phylopic.org/image/6a2f7cea-9546-4af0-a189-dd0869022ff6/) so that it better represents *Ovis aries*.
(DOCX)
