# Supplementary material for: Neural crest cell genes and the domestication syndrome: A comparative analysis of selection
Source: PLoS One. 2022 Feb 11;17(2):e0263830. doi: 10.1371/journal.pone.0263830 (PMC8836321; doi:10.1371/journal.pone.0263830)
Supplement: S1 Table — List of neural crest cell and control genes. First column indicates the names of neural crest genes investigated and the second column indicates the biochemical-molecular function of the gene. (DOCX) [file pone.0263830.s001.docx]

|  | **Neural crest cell genes** | **Biochemical-molecular function** |
| --- | --- | --- |
| **N**  **E**  **U**  **R**  **A**  **L**      **C**  **R**  **E**  **S**  **T**      **G**  **E**  **N**  **E**  **S** | *baz1b* | Chromatin regulator |
|  | *chd7* | ATP-requiring chromatin remodeler protein |
|  | *fgf8* | Growth factor/signal transduction ligand |
|  | *foxd3* | Transcription factor |
|  | *gdnf* | Glial-derived neurotrophic factor |
|  | *kit* | Receptor protein tyrosine kinase |
|  | *magoh* | Exon junction complex component |
|  | *sox 2* | HMG-transcription factor |
|  | *sox 9* | Transcription factor |
|  | *sox 10* | Transcription factor |
|  | *tcof1* | Nucleophosphoprotein |
| **C**  **O**  **N**  **T**  **R**  **O**  **L**    **G**  **E**  **N**  **E**  **S** | *commd1* | Regulator of copper homeostasis |
|  | *bace1* | Aspartic protease |
|  | *brn1* | Transcription factor |
|  | *hmga2* | Chromosomal architectural factor |
|  | *nme1* | Nucleoside diphosphate kinase |
|  | *ube2d1* | Ubiquitin conjugating enzyme |
|  | *ube2i* | Ubiquitin conjugating enzyme |
|  | *opa2* | Nuclear-encoded mitochondrial protein |
|  | *fam3a* | Cytokine-like protein |
|  | *idh1* | Isocitrate dehydrogenase |
|  | *pon1* | Paraoxonase enzyme |
