# Supplementary material for: Neural crest cell genes and the domestication syndrome: A comparative analysis of selection
Source: PLoS One. 2022 Feb 11;17(2):e0263830. doi: 10.1371/journal.pone.0263830 (PMC8836321; doi:10.1371/journal.pone.0263830)
Supplement: S2 Table — First column indicates names of domesticated animals and the second column indicates their wild counterparts. NCBI RefSeq genome accession ID is listed for each species. (DOCX) [file pone.0263830.s002.docx]

| **Domesticated animals** | **Closely related wild counterparts** |
| --- | --- |
| Dog (*Canis lupus familiars*)  Assembly: GCF_014441545.1 | African wild dog (*Lycaon pictus*)  Assembly: GCA_004216515.1 |
| Red fox (*Vulpes vulpes*)  Assembly: GCF_003160815.1 | Arctic fox (*Vulpes lagopus*)  Assembly: GCF_018345385.1 |
| Cat (*Felis catus*)  Assembly: GCF_000181335.3 | Leopard (*Panthera pardus*)  Assembly: GCF_001857705.1 |
| Horse (*Equus caballus*)  Assembly: GCF_002863925.1 | Przewalski’s horse (*Equus przewalskii*)  Assembly: GCF_000696695.1 |
| Arabian camel (*Camelus dromedarius*) Assembly: GCF_000803125.2 | Wild Bactrian camel (*Camelus ferus*)  Assembly: GCF_009834535.1 |
| Pig (*Sus scrofa*)  Assembly: GCF_000003025.6 | Chacoan peccary (*Catagonus wagneri*)  Assembly: GCA_004024745.2 |
| Goat (*Capra hircus*)  Assembly: GCF_001704415.1 | Siberian ibex (*Capra sibirica*)  Assembly: GCA_003182615.2 |
| Sheep (*Ovis aries*)  Assembly: GCF_016772045.1 | Bighorn Sheep (*Ovis Canadensis*)  Assembly: GCA_004026945.1 |
| Cattle (*Bos taurus*)  Assembly: GCF_002263795.1 | Plains bison (*Bison bison bison*)  Assembly: GCF_000754665.1 |
| Water buffalo (*Bubalus bubalis*)  Assembly: GCF_019923925.1 | African buffalo (*Syncerus caffer*)  Assembly: GCA_902825105.1 |
| Bonobo (*Pan paniscus*)  Assembly: GCF_013052645.1 | Chimpanzee (*Pan troglodytes*)  Assembly: GCF_002880755.1 |
| Rabbit (*Oryctolagus cuniculus*)  Assembly: GCF_000003625.3 | Snowshoe hare (*Lepus americanus*)  Assembly: GCA_004026855.1 |
| Guinea pig (*Cavia porcellus*)  Assembly: GCF_000151735.1 | Capybara (*Hydrochoerus hydrochaeris*)  Assembly: GCA_004027455.1 |
| House mouse (*Mus musculus*) | Ryukyu mouse (*Mus caroli*)  Assembly: GCF_900094665.1 |
| Norway rat (*Rattus norvegicus*) | African woodland thicket rat (*Grammomys surdaster*)  Assembly: GCF_004785775.1 |
