# Supplementary material for: Neural crest cell genes and the domestication syndrome: A comparative analysis of selection
Source: PLoS One. 2022 Feb 11;17(2):e0263830. doi: 10.1371/journal.pone.0263830 (PMC8836321; doi:10.1371/journal.pone.0263830)
Supplement: S1 File — All mammal silhouettes used in Fig 1 were taken from Phylopic (http://phylopic.org) and are available for use under a Public Domain license. Work under this particular license is free to use without restrictions under copyright law (https://wiki.creativecommons.org/wiki/Public_domain)”. Below you will find the link to each mammal silhouette used, indicating their Public Domain license. Of all these silhouettes, one was further modified by an author of this manuscript, Andrew O. Rubio. Modification was done to silhouettes number 19 (http://phylopic.org/image/6a2f7cea-9546-4af0-a189-dd0869022ff6/) so that it better represents Ovis aries. (DOCX) [file pone.0263830.s003.docx]

**Supplementary File: Figure 1 mammal Silhouettes**

All mammal silhouettes used in figure 1 were taken from Phylopic ([http://phylopic.org](http://phylopic.org/)) and are available for use under a Public Domain license. Work under this particular license is free to use without restrictions under copyright law (<https://wiki.creativecommons.org/wiki/Public_domain>)”. Below you will find the link to each mammal silhouette used, indicating their Public Domain license. Of all these silhouettes, one was further modified by an author of this manuscript, Andrew O. Rubio. Modification was done to silhouettes number 19 (<http://phylopic.org/image/6a2f7cea-9546-4af0-a189-dd0869022ff6/>) so that it better represents *Ovis aries*.

1. For *Felis catus* we used this image <http://phylopic.org/image/23cd6aa4-9587-4a2e-8e26-de42885004c9/>
2. For *Panthera pardus* we used this image <http://phylopic.org/image/78dbe564-bcba-4dc3-8bdc-fb95fc288580/>
3. For *Vulpes vulpes* we used this image <http://phylopic.org/image/a5e2a085-c895-4fdd-b39e-bbac8ca94d7d/>
4. For *Vulpes lagopus* we used this image <http://phylopic.org/image/8db421ba-4b42-4bd8-b948-5214ce122f57/>
5. For *Canis familiaris* we used this image <http://phylopic.org/image/6f3ebbc6-be53-4216-b45b-946f7984669b/>
6. For *Lycaon pictus* we used the image <http://phylopic.org/image/c974b08c-1189-48db-afc6-edf54356abb4/>
7. For *Equus caballus* we used this image <http://phylopic.org/image/a31e7527-3203-4233-b0da-c415cc7d1664/>
8. For *Equus przewalskis* we used this image <http://phylopic.org/image/a31e7527-3203-4233-b0da-c415cc7d1664/>
9. For *Camelus dromedarius* we used this mage <http://phylopic.org/image/d3aa9186-85bf-466d-8810-79c690b2f38d/>
10. For *Camelus ferus* we used this image <http://phylopic.org/image/e52676dd-272c-4b14-8c99-ea5dc98942e5/>
11. For *Sus scrofa* we used this image <http://phylopic.org/image/008d6d88-d1be-470a-8c70-73625c3fb4fb/>
12. For *Chacoan peccary* we used this image <http://phylopic.org/image/87047da1-b40e-4b31-8492-4db262f129f5/>
13. For *Bubalus bubalis* we used this image <http://phylopic.org/image/d36c5902-5124-41e5-a880-15b0b1d1070d/>
14. For *Syncerus caffer* we used this image <http://phylopic.org/image/65c4a9b3-dcde-4f0f-9a1f-8d71e74be9ec/>
15. For *Bos taurus* we used this image <http://phylopic.org/image/dab069c2-7664-4569-97be-11b38fe77cc2/>
16. For *Bison bison bison* we used this image <http://phylopic.org/image/d44d834e-1c32-4a08-a5d5-f30139de4450/>
17. For *Capra hircus* we used this image <http://phylopic.org/image/c39a1d31-eb36-4b62-ba4d-32e29e0e5a8d/>
18. For *Siberian ibex* we used this image <http://phylopic.org/image/52c28a94-15e2-482d-ac4d-f75ce7b09b95/>
19. For *Ovis aries* we used and modified this image. Modifications were done by Andrew Otto Rubio, an author of this manuscript. <http://phylopic.org/image/6a2f7cea-9546-4af0-a189-dd0869022ff6/>
20. For *Ovis canadensis* we used this image <http://phylopic.org/image/b7344c53-6115-49cf-836d-ae71cc3853a8/>
21. For *Pan paniscuc* we used this image <http://phylopic.org/image/37e8f97f-a9f5-42b1-a6c4-b789001c0d6c/>
22. For *Pan troglodytes* we used this image <http://phylopic.org/image/7133ab33-cc79-4d7c-9656-48717359abb4/>
23. For *Oryctolagus cuniculus* we used this image <http://phylopic.org/image/1e15411c-5394-4a9d-a209-76c8ac0c331d/>
24. For *Lepus americanus* we used this image <http://phylopic.org/image/8e61e166-11f4-4377-a923-9b5b597b6eba/>
25. For *Cavia porcellus* we used this image <http://phylopic.org/image/75836dad-906e-4066-8518-ab60f0f96b73/>
26. For *Hydrochoerus hydrochaeris* we used this image <http://phylopic.org/image/097336ef-db5f-4edc-a2e4-2c29a10aa512/>
27. For *Mus musculus* we used this image <http://phylopic.org/image/c8f71c27-71db-4b34-ac2d-e97fea8762cf/>
28. For *Mus caroli* we used this image <http://phylopic.org/image/6b2b98f6-f879-445f-9ac2-2c2563157025/>
29. For *Rattus norvegicus* we used this image <http://phylopic.org/image/570c7d9e-e6d1-46f5-b165-988981bfc5f6/>
30. For *Grammomys surdaster* we used this image <http://phylopic.org/image/a460430b-472b-4018-ba03-6b8eeb57fa5c/>
